# In silico analysis of Triphala-derived polyphenols as inhibitors of TIR–TIR homodimerization in the inflammatory pathway

**DOI:** 10.3389/fbinf.2025.1565700

**Published:** 2025-05-29

**Authors:** Durgadevi Rajendran, Nalini Easwaran

**Affiliations:** School of Bio Sciences and Technology, Vellore Institute of Technology, Vellore, India

**Keywords:** nuclear factor kappa-light-chain-enhancer of activated B cells, myeloid differentiation primary response gene 88, Triphala, TIR–TIR homodimerization, docking, molecular dynamics simulation

## Abstract

Downstream signaling of the nuclear factor kappa-light-chain-enhancer of activated B cells (NF-κB) pathway is mediated by the adaptor protein myeloid differentiation primary response gene 88 (*MyD88*). The TIR domain present in *MyD88* plays a pivotal role in regulating the expression of pro-inflammatory cytokines. Although synthetic drugs, including M20 and TJ-M2010-5, have been studied to mitigate the overexpression of *MyD88*, their prolonged usage is known to cause adverse side effects, highlighting the need for a safer, risk-free alternative. An Ayurvedic formulation named Triphala, which is rich in polyphenols and traditionally used to treat various ailments, was selected for this investigation. Although polyphenols are gaining attention as anti-inflammatory agents, their precise mode of action remains insufficiently understood. Previous studies have explored the anti-inflammatory properties of Triphala in a broad spectrum, but this study notably focuses on the interactions of Triphala-derived polyphenols with the TIR domain of the MyD88 adaptor protein in the NF-κB signaling pathway. This study employs computational docking and a molecular dynamics (MD) simulation to study the interaction and stability of the polyphenols with the target protein. The polyphenols were virtually docked to the TIR domain of MyD88 using AutoDock tools 1.5.7. Among them, the top three protein–polyphenol complexes with the highest binding affinities were selected and subjected to MD simulation for 200 ns to evaluate their interaction properties in detail. The findings of the MD simulation corroborated the docking results, showing that two complexes (protein–punicalagin and protein–chebulagic acid) demonstrated better interaction patterns. The MD trajectory revealed that polyphenol binding enhanced the stability of the target protein, as indicated by lower root-mean-square deviation (RMSD) (∼0.25 nm), solvent accessible surface area (SASA) (∼96.848–100.666 nm^2^), and stabilized radius of gyration (Rg) (∼1.50–1.53 nm) values for punicalagin and chebulagic acid complexes compared to the reference complex. Our findings have supported the hypothesis that Triphala polyphenols may interact with the TIR domain of MyD88, thereby inhibiting the production of inflammatory cytokines. This study provides a combination of computational validation of specific molecular targets and mechanistic insights into the anti-inflammatory potential of Triphala-derived polyphenols.

## Introduction

Myeloid differentiation primary response gene 88 (*MyD88*) is a cytosolic critical adaptor protein that plays a crucial role in the downstream signaling of the innate immune response (Di Padova et al., 2018). Although there is a vast array of mechanisms and proteins that contribute to the activation of the innate immune response, MyD88 acts as a central mediator in triggering the process (Olson et al., 2015). It facilitates the downstream signaling of the nuclear factor kappa-light-chain-enhancer of activated B cells (NF-κB) pathway and releases pro-inflammatory cytokines. Imbalance of MyD88 triggers a wide spectrum of inflammatory diseases, highlighting its significance in the homeostasis of innate immunity. MyD88 is predominantly activated by Toll-like receptors (TLRs) (except TLR3). TLRs are the major class of pathogen recognition receptors (PRRs) that can recognize the pathogen-associated molecular patterns (PAMPs) present in the microbe (Wang et al., 2014). PAMPs such as lipopolysaccharides (LPS) can bind to the leucine-rich repeat (LRR) motifs of TLRs. This interaction promotes the dimerization of the Toll/interleukin-1 receptor (TIR) domains, subsequently recruiting the TIR domain-containing adaptor proteins, including MyD88 (Clabbers et al., 2021; Saikh, 2021). MyD88 consists of three domains, namely, the N-terminal death domain (DD), the C-terminal Toll/interleukin-1 receptor (TIR) domain, and the intermediate domain (INT). The TIR domain (155–296 aa) facilitates the interaction of MyD88 with the TIR domain of TLR or IL-1R receptors to form a higher-order complex, thereby recruiting additional TIR domains in the vicinity and propagating the downstream signaling cascade. This strongly suggests that TIR–TIR homodimerization may be a viable drug target to inhibit inflammation. Previous studies have focused on developing mimetic small-molecule inhibitors that target the BB-loop of the TIR domain to elucidate the inhibitory mechanism of MyD88 homodimerization (Loiarro et al., 2005; Saikh, 2021).

Several studies have demonstrated that synthetic analogs such as ST2825 effectively inhibit the dimerization of MyD88 by binding to the BB-loop within the TIR domain, thereby disrupting NF-κB-mediated signal transduction in pro-inflammatory cytokine production (Loiarro et al., 2007). Furthermore, ST2825 has been studied to reduce oxidative stress by attenuating the production of reactive oxygen species (ROS) and suppressing NLRP3 inflammasome activation, ultimately inhibiting caspase-1 inflammatory pathways (Zhang et al., 2022). Additionally, TJ-M2010-5 is a mimetic drug designed to interact with the TIR domain of MyD88 to inhibit homodimerization. It binds to the αE, βD, βC, αA, DD, and EE loops but not to the BB-loop of the TIR domain (Xie et al., 2016). However, studies show that this drug has some key limitations and is more effective when administered prophylactically rather than therapeutically (Zou et al., 2023). M20 is another small-molecule inhibitor designed to reduce MyD88 homodimerization by binding to the hydrophobic (αC′–βD–αD) pocket of the TIR domain (Song et al., 2021). These synthetic drugs need to be investigated further for their limitations, mainly regarding their toxicity associated with prolonged usage. In contrast, ancient medicinal systems such as Siddha and Ayurveda provide alternative therapeutic approaches that play a rescuing role in such cases. These natural medicines have potentially lower toxicity profiles and are rich in bioactive compounds that exhibit strong anti-inflammatory properties. Triphala is a well-known ancient polyherbal formulation made up of three fruits, namely, *Terminalia chebula*, *Terminalia bellerica*, and *Phyllanthus emblica*, in a ratio of 1:1:1. This formulation is rich in bioactive compounds such as polyphenols, flavonoids, and tannins. They have been found to play a positive role in the treatment of various inflammatory diseases such as arthritis, cancer, diabetes, and gastrointestinal tract disorders. (Shanmuganathan and Angayarkanni, 2018). Studies have demonstrated that Triphala can hamper the production of pro-inflammatory mediators such as interleukin-6 (IL-6), interleukin-1 beta (IL-1β), and tumor necrosis factor-alpha (TNF-α) by inhibiting the NF-κB signaling pathway (Kalaiselvan and Rasool, 2016). All the above-mentioned studies have explored the general immunomodulatory effects of Triphala. However, no *in silico* or experimental evidence is currently available for Triphala-derived polyphenol-mediated inhibition of MyD88. Our study provides insights into the direct molecular interactions between Triphala polyphenols and the MyD88 adaptor protein, unveiling the underlying mechanism behind the immunomodulatory properties of these polyphenols through computational screening approaches.

## Materials and methods

### Database and software

The databases and software applications used in this study include RCSB PDB (Protein Data Bank) (https://www.rcsb.org), PubChem (https://pubchem.ncbi.nlm.nih.gov/), ChemSpider (http://www.chemspider.com), Open Babel version 3.1.1 (https://github.com/openbabel/openbabel/releases), pkCSM webserver (https://biosig.lab.uq.edu.au/pkcsm/prediction), PyMOL 3.0.3 (https://www.pymol.org), AutoDock tools 1.5.7 (https://ccsb.scripps.edu/mgltools/downloads/), Swiss-PDB Viewer version 4.1.0 (https://spdbv.unil.ch/download/binaries/SPDBV_4.10_PC.zip), AutoDock Vina version 1.1.2 (https://vina.scripps.edu/wp-content/uploads/sites/55/2020/12/autodock_vina_1_1_2_linux_x86.tgz), LigPlot + v.2.2 (https://www.ebi.ac.uk/thornton-srv/software/LigPlus/), and GROMACS version 2023.1 (https://zenodo.org/records/7852175/files/gromacs-2023.1.tar.gz?download=1).

### Preparation of the protein

The crystal structure of the TIR domain of the adaptor protein MyD88 (PDB ID: 4DOM) was retrieved from the RCSB PDB database, which contains 151 amino acids (Clabbers et al., 2021). The structure was determined by X-ray diffraction to a resolution of 2.30 Å. Heteroatoms, water molecules, and undesired ligand molecules were removed using PyMOL. Missing atoms were fixed using Swiss-PDB Viewer version 4.1.0. Using AutoDock tools 1.5.7, polar hydrogens and Kollman charges were added to stabilize the protein structure and were saved in the PDBQT file format for molecular docking (Hu et al., 2024).

### Ligand preparation

A total of 33 Triphala-derived bioactive compounds with strong interaction properties toward the target protein were selected based on a literature survey. For instance, all the selected ligands have been frequently explored for their strong anti-inflammatory, anticancer, and antioxidant properties. The 3D structures of these ligands were retrieved from the PubChem and ChemSpider databases (Tarasiuk et al., 2018; Prasad and Srivastava, 2020; Rudrapal et al., 2022; Wang et al., 2023). Energy minimization of all the ligands was performed by applying Merck Molecular Force Field 94 (MMFF94) to obtain stable conformations, as MMFF94 was specifically developed to provide accurate intermolecular geometries for small organic molecules. All the ligands were then converted to the PDBQT format for molecular docking (Murugesan et al., 2021; Olaokun et al., 2022).

### Molecular docking

The processed ligands were subjected to docking against the TIR domain of MyD88 using AutoDock Vina version 1.1.2. In this study, the ligands that were energy minimized were given as the input file, and the prepared protein was given as the target file. To perform high-throughput molecular docking, a Perl script was used containing multiple ligand files to execute AutoDock Vina (Eberhardt et al., 2021). The blind docking covers the whole protein, and the center of the grid box was precisely positioned at the coordinates X: 17.67, Y: 1.496, and Z: 12.318 with a size range of X: 58 Å, Y: 54 Å, and Z: 46 Å. This configuration facilitates the sampling of the entire protein surface by the ligands to explore all the potential binding pockets. A maximum of 10 docking conformers were set, from which the most favorable binding energies were selected, and the root-mean-square deviation (RMSD) values were saved (Aliye et al., 2021; Eberhardt et al., 2021). Complexes of the saved confirmations of ligands and target proteins were written using PyMOL 3.0.3. Non-covalent interactions of the complexes were studied using LigPlot+ (Laskowski and Swindells, 2011). Furthermore, an MD simulation along with calculations of binding free energies was carried out for these complexes to understand the dynamic properties involved in the interaction and stability of the protein–ligand complexes (Rudrapal et al., 2022).

### Molecular dynamics simulation

An MD simulation was performed for the complexes with the top three binding affinities, the reference drug (TJ-M2010-5), and the MyD88 protein alone using GROningen MAchine for Chemical Simulations (GROMACS) version 2023.1 (Abraham et al., 2015; Rudrapal et al., 2022). The CHARMM (Chemistry at HARvard Macromolecular Mechanics) force field was employed for the MD simulation of all the complexes due to its robust parameterization and reliability in modeling biomolecular systems, including small molecules and phytochemicals. The topology file for the target protein was prepared using GROMACS, and the target ligands were prepared using CGenFF (Vanommeslaeghe et al., 2010). The periodic boundary was set using a rectangular box, and the water box with solute molecules surrounded by the solvent was extended to 10 Å. Bond constraints were handled using the LINCS algorithm. All the complexes were solvated using the TIP3P water model, followed by sodium and chloride ions being added for neutralization (Ghosh et al., 2021; Murugesan et al., 2021). Energy minimization was carried out for all the complexes using the steepest descent algorithm. Equilibration was carried out under isothermal–isochoric (NVT) and isothermal–isobaric (NPT) conditions for 0.3 ns each (Rudrapal et al., 2022). The systems were then subjected to an MD simulation under a pressure of 1 atm and a temperature of 300 K for 200 ns (duplicates were run to ensure reproducibility). Here, the leapfrog algorithm was used at 2 fs for integrating the motion equation of atoms. Finally, the average distance between the atoms of the overlying protein–ligand complex was studied by RMSD. Deviations in the positions of the atoms were studied by root-mean-square fluctuation (RMSF). The overall size and compactness were studied using the radius of gyration (Rg), and the accessibility of the surface area to the solvent molecules was quantified by solvent accessible surface area (SASA). All the above four parameters corroborated the stability of the target protein in the presence of ligands, suggesting the sustainability of the interaction. The non-covalent associations (H-bonds) were calculated to evaluate the stability and specificity of the molecular interactions using GROMACS scripts (Ghosh et al., 2021; Rudrapal et al., 2022).

### Molecular mechanics Poisson–Boltzmann surface area

Binding free energy is the energy that tends to be released when a protein and a ligand form a complex, and it can be calculated using the molecular mechanics Poisson–Boltzmann surface area (MM-PBSA) method. MM-PBSA helps in understanding the thermodynamics and kinetics involved in the processes of molecular binding. Dielectric constants are the key parameters to calculate MM-PBSA, which enables the accurate estimation of the electrostatic contribution to the reaction medium. An internal dielectric constant (indi) of 4.0 was adopted to approximate the electric polarizability of the solute (protein), whereas an external dielectric constant (exdi) of 80.0 was set to simulate the environment of bulk water at room temperature (Hou et al., 2011). Snapshots were recorded at an interval of 0.1 ns (100 picoseconds) throughout the 200 ns simulation. The frames of the trajectory used for the MM-PBSA calculation were extracted using the parameter nstxtcout = 50,000, corresponding to one frame every 0.1 ns. A total of 2,000 frames were taken to cover the whole trajectory of 200 ns rather than focusing on a segment of the trajectory. The parameters, such as changes in bond energies, angles, electrostatics, and van der Waals and solvation-free energy, which is the sum of polar and nonpolar solvation energies, were calculated. The overall binding free energy was calculated using the following formula (Kumari et al., 2014; Murugesan et al., 2021; Rudrapal et al., 2022):

$$\Delta G_{\text{bind}}=\Delta G_{\text{complex}}-\left(\Delta G_{\text{protein}}+\Delta G_{\text{ligand}}\right),$$

where ΔG_bind_ is the binding free energy between the ligand and protein, ΔG_complex_ is the binding free energy released by the protein–ligand complex, ΔG_protein_ is the binding free energy of the unbound protein, and ΔG_ligand_ is the binding free energy of the unbound ligand.

### Drug-likeness and pharmacokinetic profile

Drug-likeness is a qualitative property that indicates whether a drug molecule can act as a potential drug. The rule of five (Ro5) or Lipinski’s rule of five provides a set of guidelines to evaluate the drug-likeness of oral drugs (Lipinski, 2004). These rules provide criteria for certain molecular properties that play a major role in pharmacokinetic profiles, such as absorption, distribution, metabolism, and excretion (ADME). In this study, SwissADME and pkCSM were used to study drug-likeness, physicochemical properties, and pharmacokinetics (Pires et al., 2015; Daina et al., 2017; Ghosh et al., 2021).

## Results

### Molecular docking

**FIGURE 1:**
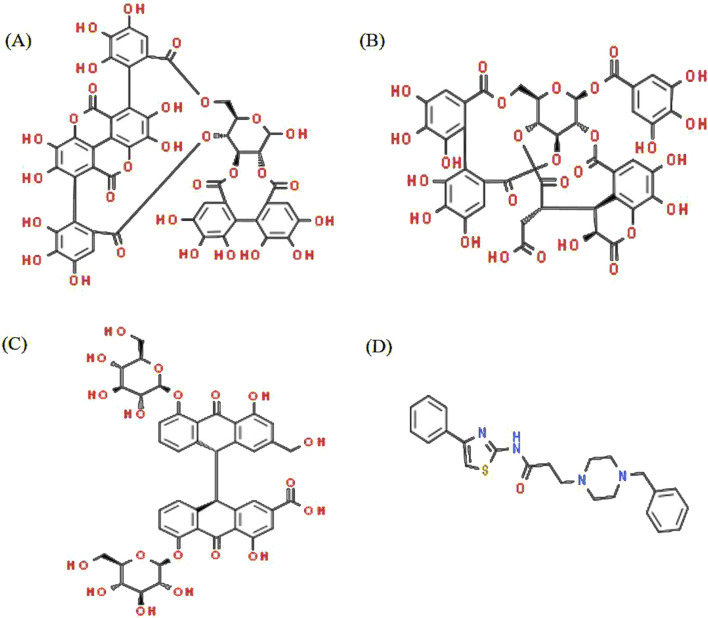
Structure of Triphala ligands with the top three binding affinities and the reference compound. **(A)** Punicalagin, **(B)** chebulagic acid, **(C)** sennoside C, and **(D)** TJ-M2010-5.

**FIGURE 2:**
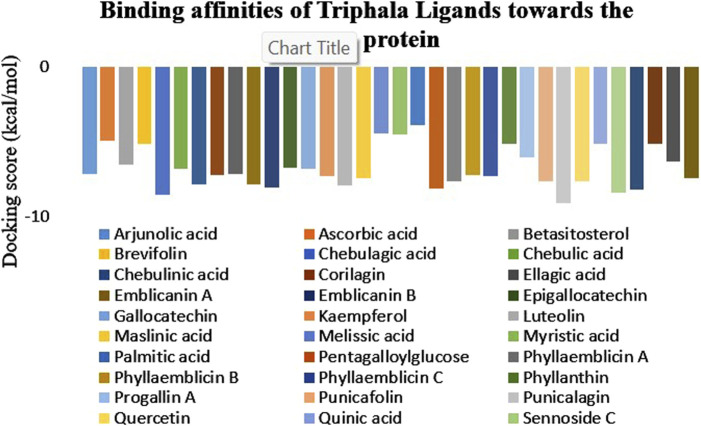
Graphical representation of the binding affinities of Triphala-derived ligands against the target protein. The graph illustrates the docking scores of various ligands, wherein the maximum negative score represents the highest binding affinity for the target protein.

**FIGURE 3:**
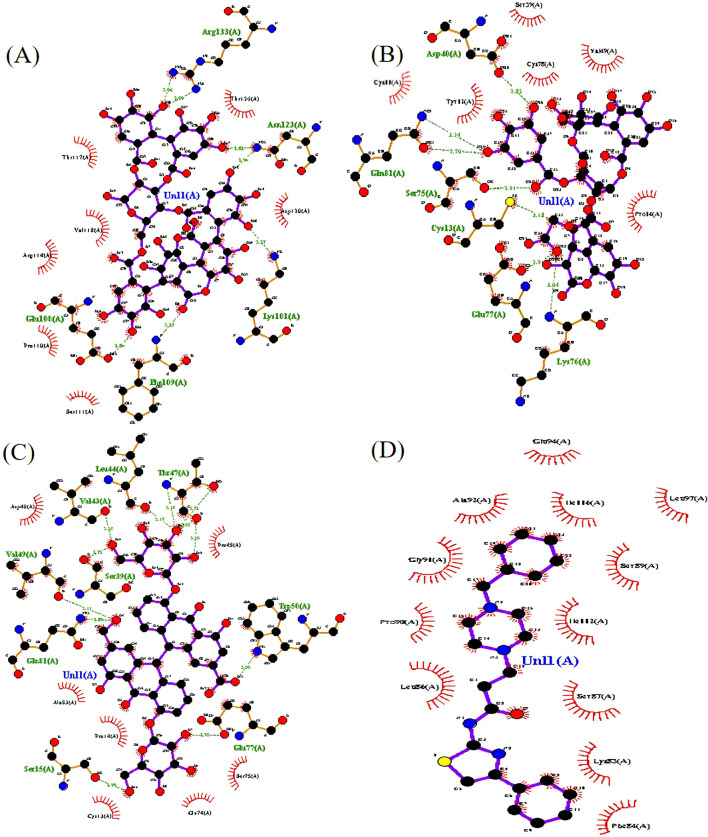
The top three protein–ligand complexes with hydrogen bond interactions (studied using LigPlot + v.2.2). **(A)** Interaction of punicalagin with the target protein. **(B)** Interaction of chebulagic acid with the target protein. **(C)** Interaction of sennoside C with the target protein. **(D)** Interaction of TJ-M2010-5 with the target protein.

A list of 33 polyphenols derived from the Ayurvedic formulation Triphala were taken and computationally docked with the crystal structure of the TIR domain of the MyD88 protein to evaluate their interaction and binding affinities (Tarasiuk et al., 2018; Prasad and Srivastava, 2020; Wang et al., 2023). TJ-M2010-5, a synthetic anti-inflammatory drug, was taken as a reference molecule (Xie et al., 2016). Binding affinities were assessed based on the docking scores, expressed in kcal/mol (Song et al., 2021; Hu et al., 2024). All the polyphenols exhibited favorable interaction patterns, and few polyphenols, such as chebulagic acid, chebulinic acid, emblicanin B, emblicanin A, luteolin, malinic acid, maslinic acid, pentagalloylglucose, punicalagin, punicafolin, quercetin, sennoside C, and sennoside E, exhibited good binding affinities toward the target protein compared to the reference molecule. In particular, punicalagin (−9.1 kcal/mol), sennoside C (−8.4 kcal/mol), and chebulagic acid (−8.5 kcal/mol) showed the highest binding affinities compared to the reference drug TJ-M2010-5 (−7.4 kcal/mol), suggesting that the polyphenols have better interaction properties with the target protein than the reference drug (Table 1) (Figures 1, 2). However, the practical application of polyphenols as inhibitors of MyD88 depends on several additional factors such as molecular interaction, stability, and binding free energy, which were also elaborately investigated in the present study. The non-covalent interactions between the protein–ligand complexes were examined in detail using LigPlot + V.2.2. In this study, punicalagin was observed to interact with Arg133, Asn123, Lys101, Phe109, and Glu108 amino acid residues present in the TIR domain of MyD88 through seven hydrogen bonds (reversible non-covalent bonds). Similarly, chebulagic acid was observed to have established seven hydrogen bonds with Asp40, Gln81, Ser75, Cys13, Glu77, and Lys76 amino acid residues of the target protein. Notably, sennoside C established 11 hydrogen bonds with Trp50, Glu77, Ser15, Gln81, Ser39, Val49, Val43, Leu44, and Thr47 acid residues of the target protein. In contrast, the reference drug TJ-M2010-5 did not form any hydrogen bonds with the target protein, which shows that the interaction profile of the reference drug was weaker than that of Triphala-derived polyphenols (Table 2) (Figure 3).

**TABLE 1:** Binding affinity or docking scores of different Triphala polyphenols and the reference compound with the TIR domain of the MyD88 protein.

S.No	Ligand	Docking score/binding affinity (kcal/mol)
1	Arjunolic acid	−7.1
2	Ascorbic acid	−4.9
3	Betasitosterol	−6.5
4	Brevifolin	−5.1
5	**Chebulagic acid**	**−8.5**
6	Chebulic acid	−6.8
7	Chebulinic acid	−7.8
8	Corilagin	−7.2
9	Ellagic acid	−7.1
10	Emblicanin A	−7.8
11	Emblicanin B	−8
12	Epigallocatechin	−6.7
13	Gallocatechin	−6.8
14	Kaempferol	−7.3
15	Luteolin	−7.9
16	Maslinic acid	−7.4
17	Melissic acid	−4.4
18	Myristic acid	−4.5
19	Palmitic acid	−3.9
20	Pentagalloylglucose	−8.1
21	Phyllaemblicin A	−7.6
22	Phyllaemblicin B	−7.2
23	Phyllaemblicin C	−7.3
24	Phyllanthin	−5.1
25	Progallin A	−6
26	Punicafolin	−7.6
27	**Punicalagin**	**−9.1**
28	Quercetin	−7.6
29	Quinic acid	−5.1
30	**Sennoside C**	**−8.4**
31	Sennoside E	−8.2
32	Syringic acid	−5.1
33	Tannic acid	−6.3
34	**TJ-M2010-5**	**−7.4**

Note: the more negative the docking score, the higher the binding affinity for the ligand with the target protein.

Bolded values indicate the highest binding affinities (most negative docking scores) of the tested compounds against the target protein.

**TABLE 2:** Formation of hydrogen bonds between the ligands and the target protein with their interacting residues.

Ligands	No. of H bonds formed by the ligands with the protein	Interacting amino acid residues in the protein
Punicalagin	7	Arg133, Asn123, Lys101, Phe109, and Glu108
Chebulagic acid	7	Asp40, Gln81, Ser75, Cys13, Glu77, and Lys76
Sennoside C	11	Trp50, Glu77, Ser15, Gln81, Ser39, Val49, Val43, Leu44, and Thr47
TJ-M2010-5	0	Nil

### Molecular dynamics simulation

**FIGURE 4:**
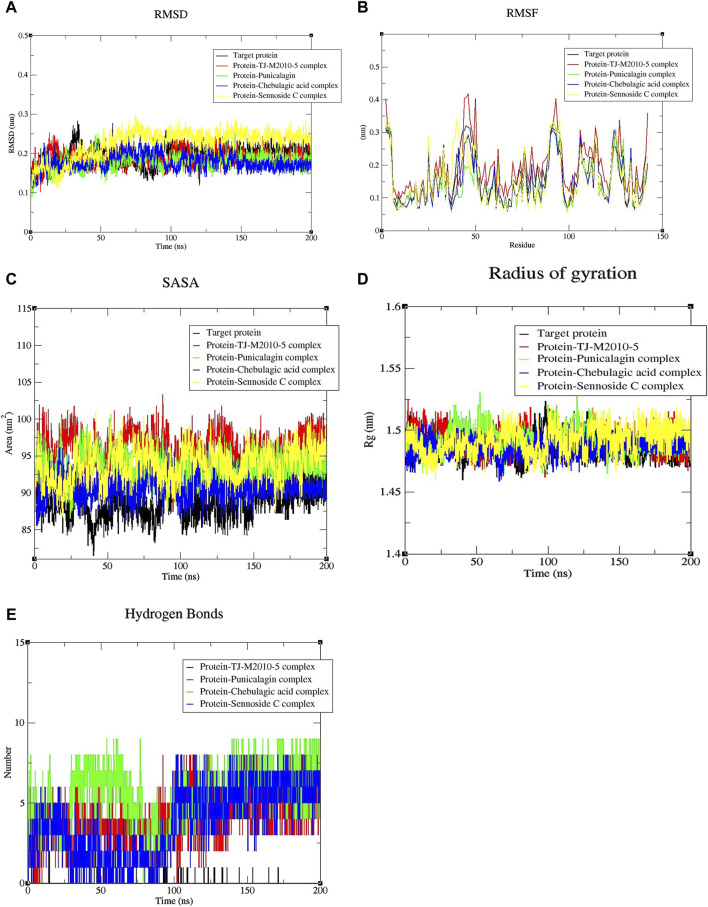
Graphical plots for the top three complexes, the reference complex, and the target protein alone. **(A)** RMSD shows the interaction, stability, and conformational changes of the molecular structures of the different complexes (p-value = 0.0227). **(B)** RMSF shows the positional fluctuation of individual residues of the protein in different complexes (p-value = 0.0118). **(C)** SASA shows the degree of the protein surface exposed to the solvent present in the medium (p-value = 0.0130). **(D)** Radius of gyration (Rg) measures the compactness, folding, and unfolding of the protein structure in different complexes (p-value = 0.0465). **(E)** H-bond shows the number of H-bonds formed in the complexes during dynamic conditions (p-value = 0.0118). All of the above-mentioned p-values are <0.05, indicating statistical significance. Note: one-way ANOVA with a Brown–Forsythe test was used for comparing each parameter individually in duplicate for significance.

The top three complexes with higher binding affinities, the reference complex, and the target protein were studied for their stability, interaction, and confirmation in detail by performing an MD simulation for 200 ns (Ghosh et al., 2021; Aljarba et al., 2022). The stability of the complexes was assessed by studying key parameters, including RMSD, RMSF, SASA, Rg, and the hydrogen bonds formed. Graphical representations for these parameters were generated using the Xmgrace 2D plotter (Ghosh et al., 2021; Rudrapal et al., 2022; Vieira et al., 2023). RMSD was employed to study the conformational stability and structural integrity of the molecules present in the protein–ligand complexes (Ghosh et al., 2021; Rudrapal et al., 2022). The RMSD of the target protein was gradually increased from 0 to 33.5 ns and reached a peak of 0.28 nm, followed by a sudden decrease. Subsequently, the RMSD values showed continuous fluctuations and reached up to 0.27 nm at 58.4 nm, before a gradual decrease until 92.8 ns. Following that, the RMSD stabilized with minor fluctuations; the protein backbone was normalized after 149.7 ns and remained stable throughout the simulation with minor fluctuations. The RMSD profile of the protein–TJ-M2010-5 complex was more variable in the beginning, reaching up to 0.25 nm at 13.4 ns, followed by minor fluctuations throughout the MD simulation for 200 ns, indicating the structural instability of the complex. The protein–punicalagin complex first displayed significant fluctuations in RMSD and reached a maximum value of 0.25 nm at 48.2 ns and 145.8 ns. After that, the complex stabilized with some minor deviations. The protein–chebulagic acid complex exhibited oscillations in RMSD from 0 to 107.7 ns and reached a maximum value of 0.25 nm at 69.9 nm. Subsequently, the values stabilized with slight deviations until 145.8 ns and remained stable throughout the 200 ns. The RMSD exhibited by the protein–sennoside C complex was the highest of all the other complexes and the target protein, which reached a maximum value of 0.29 nm at 74.8 ns. The trajectory showed continuous oscillations until 146.8 ns, followed by the complex being partially stabilized with minor fluctuations (Figure 4A). Overall, punicalagin and chebulagic acid stabilized the backbone of the target protein compared to other ligands.

RMSF analysis helps identify regions of increased flexibility and stability within proteins. The RMSF profile of the target protein varied significantly between 2–5, 40–50, 88–96, 105–117, and 123–129 residues of amino acids. For the protein–TJ-M2010-5 complex, major fluctuations in the RMSF were found between the 2–5, 43–50, 86–96, 105–117, 123–129, and 141–142 residues of amino acids. The complex showed more fluctuations in its residues than the target protein. For the protein–punicalagin complex, major fluctuations were observed between the 2–5, 50, 89–95, 106–107, 112–115, 123–125, and 129 amino acid residues. The complex exhibited fewer fluctuations compared to the target protein, showing the stabilizing effect of punicalagin when bound to the target protein. Similarly, the protein–chebulagic complex displayed fluctuations between 2–5, 41–50, 90–96, and 123–127 amino acid residues, which have significantly lower RMSF than the target protein. The protein–sennoside C complex exhibited fluctuations between the 2–5, 38–50, 89–95, 114–115, 123–126, and 142 amino acid residues. This complex showed lower fluctuations than the target protein, although the fluctuations were slightly higher than those of punicalagin and chebulagic acid complexes (Figure 4B).

SASA was analyzed to gain insights into the structural and functional properties of biomolecules by quantifying the protein surface exposed to the solvent present in the environment. Effective ligand binding was expected to minimize the solvent accessible surface area of the protein. The target protein exhibited a maximum SASA value of 95.450 nm^2^. In contrast, the reference complex showed a maximum SASA value of 103.325 nm^2^, which is higher than that of the target protein, indicating that ligand binding was disturbed by MD simulation. The maximum SASA values recorded for the protein–punicalagin, protein–chebulagic acid, and protein–sennoside C complexes were 100.666, 96.848, and 101.003 nm^2^, respectively. Subsequently, the values were reduced during a further simulation for 200 ns, suggesting potential protein stabilization by ligands over the course of the MD simulation (Figure 4C). Overall, punicalagin and chebulagic acid complexes exhibited lower SASA values than the reference drug. On the other hand, sennoside C showed elevated SASA values, similar to the reference drug. This data trend shows a strong correlation with the docking results, in which punicalagin and chebulagic acid exhibited the lowest binding energies, which ultimately implies stable binding with the target protein. Rg shows the compactness of the molecular system, thereby providing insights into the stability, folding, and unfolding conformational changes in the protein. The Rg of the target protein showed minor fluctuations initially and gradually increased, reaching a maximum of 1.52 nm on 98.4 ns. Subsequently, the system was stabilized after 148.4 ns and remained the same throughout the simulation. Conversely, the Rg value of the reference complex displayed continuous fluctuations throughout the simulation and reached a maximum of 1.52 nm at 98.4 ns. This suggests that ligand binding negatively influences the rigidity of the protein structure. Initially, the Rg values of the punicalagin, chebulagic acid, and sennoside C complexes oscillated and reached maximums of 1.53, 1.50, and 1.51 nm, respectively. Following that, the Rg values of punicalagin and chebulagic acid complexes were stabilized after 147.7 ns and 148.8 ns, respectively. Notably, both punicalagin and chebulagic acid complexes exhibited better structural rigidity than the other complexes (Figure 4D). Furthermore, the stability of the complexes was assessed by studying the number of intermolecular hydrogen bonds, which helps maintain the complex intact. In this study, the reference complex did not form any hydrogen bonds throughout the simulation, indicating an unstable binding interaction. The H-bonds formed by the punicalagin, chebulagic acid, and sennoside C complexes varied between 1–7, 1–9, and 1–8, respectively, throughout the simulation (Figure 4E). Notably, the chebulagic acid and sennoside C complexes displayed an increased and decreased number of H-bonds in post-simulation compared with the binding energy from the docking results. An increase in bond number represents the additional conformational states that were explored by the ligand, thereby enhancing its interaction and stability under dynamic conditions, whereas a decline in bond number suggests a weaker binding of the ligand. The statistical significance tests were carried out individually for all the parameters using one-way ANOVA with the Brown–Forsythe test, and all the parameters were statistically significant (p-values are <0.05).

### Molecular mechanics Poisson–Boltzmann surface area

**FIGURE 5:**
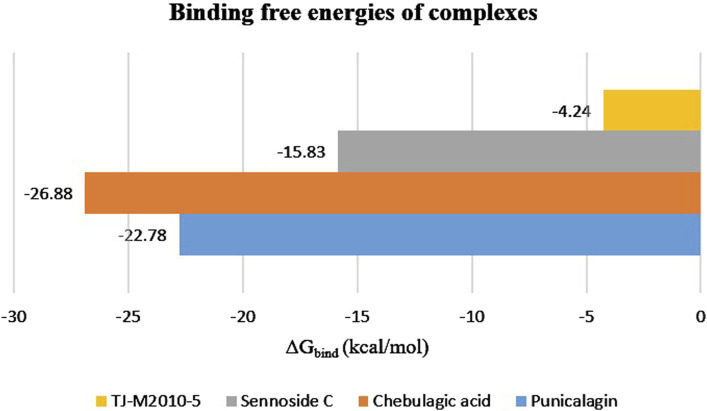
MM-PBSA analysis of the 200°ns trajectory to estimate the binding free energies of the top three protein–ligand complexes, along with the reference complex.

Binding free energy (ΔG_bind_) was computed using the sum of SASA, van der Waals, electrostatic energy, and polar and non-polar solvation energies. MM-PBSA helps in comparing the binding affinities of different proteins and ligands. A more negative ΔG_bind_ indicates a stronger binding affinity between the ligand and the target protein. The results of MM-PBSA provided insights into the energy contributed to the binding of the target protein and ligands. The average energy exhibited by the protein–punicalagin complex was −1,974.13 kcal/mol, whereas the protein and ligand alone displayed −2,320.87 kcal/mol and 369.52 kcal/mol, respectively, leading to a total ΔG_bind_ of −22.78 kcal/mol. Similarly, the protein–chebulagic acid complex exhibited an average energy of −2,234.98 kcal/mol, with the unbound protein and ligand displaying −2,353.13 kcal/mol and 145.02 kcal/mol, respectively, yielding the total ΔG_bind_ of −26.88 kcal/mol. The protein–sennoside C complex showed an average energy of −2,092.39 kcal/mol, the protein alone exhibited −2,316.08 kcal/mol, the ligand alone exhibited 23.51 kcal/mol, and the total ΔG_bind_ was −15.83 kcal/mol. For comparison, the reference complex was also analyzed for its ΔG_bind_, which was approximately −4.24 kcal/mol. The energy displayed by the protein–TJ-M2010-5 complex was −2,374.9 kcal/mol, the protein alone exhibited −2,334.55 kcal/mol, and the ligand alone exhibited −36.12 kcal/mol. Overall, punicalagin and chebulagic acid, among the other ligands, demonstrate the most favorable thermodynamic integrity. The MM-PBSA values of the polyphenols were consistent with the SASA and H-bond results, which suggest a reduction in the exposure of the protein surface toward the solvent upon ligand binding and stable intermolecular hydrogen bonding throughout the MD simulation of 200 ns (Table 3) (Figure 5).

**TABLE 3:** MM-PBSA of the top three complexes along with the reference complex.

Energy	Complex			
	Punicalagin	Chebulagic acid	Sennoside C	TJ-M2010-5
Complex (ΔG_complex_)	−1974.13	−2,234.98	−2092.39	−2,374.91
Target protein (ΔG_protein_)	−2,320.87	−2,353.13	−2,316.08	−2,334.55
Ligands (ΔG_ligand_)	369.52	145.02	23.51	−36.12
Total (ΔG_bind_)	−22.78	−26.88	−15.83	−4.24

### Drug likeness and pharmacokinetic profile

SwissADME and pkCSM were utilized to study Lipinski’s rule of five and the pharmacokinetic properties of the ligands (Ghosh et al., 2021). The synthetic reference drug exhibited no violations of Lipinski’s rule of five, whereas all three Triphala-derived ligands showed three violations: 1. molecular weight exceeding 500 Da, 2. nitrogen-oxygen bonds exceeding 10, and 3. nitrogen–hydroxyl bonds surpassing 5. Specifically, the molecular weights of punicalagin, chebulagic acid, and sennoside C were 1,084.72, 954.66, and 848.76 Da, respectively. All three polyphenols exceeded the threshold for nitrogen–oxygen bonds and nitrogen–hydroxyl bonds up to 10 and 5, respectively. The normal bioavailability score range was 0–10, and all the polyphenols showed poor bioavailability (0.11–0.17), while the reference compound exhibited a significantly higher bioavailability score of 0.55 (Martin, 2005; Daina et al., 2017). All the polyphenols displayed a water solubility of −2.892, while the reference compound had a solubility of −3.812, suggesting that the ligands ranged from moderately soluble to soluble (Daina et al., 2017). Further pharmacokinetic evaluation indicated that punicalagin, chebulagic acid, and TJ-M2010-5 demonstrated good intestinal absorption (100%, 93.835%, and 91.865%, respectively), while sennoside C showed a very poor intestinal absorption rate of 0% (Zhao et al., 2002). Even though sennoside C exhibited significant binding affinity toward the TIR domain, this major limitation, the lack of intestinal absorption, reduces its oral efficacy, and it can be overcome by advanced drug delivery platforms such as nanoparticle encapsulation (Sun et al., 2024).

The blood–brain barrier (BBB) membrane permeability revealed that TJ-M2010-5 exhibited a permeability of 0.287 log BB, whereas Triphala polyphenols showed very poor permeability (<-1) compared to the reference compound. Furthermore, none of the ligands met the range of −0.89 to 3.32 log mL/min/kg in total clearance (Zhivkova and Doytchinova, 2013). Cytochrome P450 (CYP450) enzymes play a crucial role in drug metabolism, particularly with CYP2D6/CYP3A4. An ideal drug should function as a CYP substrate for proper metabolic processing. The reference drug functions as a CYP substrate, whereas all the other tested ligands failed to act as a CYP substrate. Additionally, a drug candidate should not inhibit CYP450 enzymes. All the polyphenols did not show any inhibitory properties toward CYP450 enzymes; in contrast, the reference drug demonstrated inhibitory effects over these enzymes. Organic cation transporter 2 (OCT2) is a renal transporter that plays a crucial role in the excretion of drugs. An ideal drug should serve as an OCT2 substrate for proper excretion. However, all three polyphenols failed to exhibit the property of being OCT2 substrates; in contrast, the reference drug acted as an OCT2 substrate. Toxicity assessments showed that the Triphala-derived ligands did not exhibit hepatotoxicity or AMES (mutagenic) toxicity, whereas the reference compound displayed hepatotoxicity. These computational data align with the previously reported *in vivo* toxicity studies conducted in Sprague–Dawley rats, which suggest that the Triphala extract administration is not associated with hepatotoxicity. This was evidenced by the normal serum levels of hepatic biomarkers, including aspartate aminotransferase (AST), alanine aminotransferase (ALT), and alkaline phosphatase (ALP) levels (Arpornchayanon et al., 2022). The predicted maximum tolerated doses for punicalagin, chebulagic acid, and sennoside C were 0.438, 0.438, and 0.458 log mg/kg/day, respectively, while the reference drug had a higher tolerated dose of 0.505 log mg/kg/day (Pires et al., 2015). Rat oral acute and chronic toxicities were also evaluated with the pkCSM predictive model; the lethal dose LD50 for all the polyphenols was commonly in the acute toxicity range of 2.482 mol/kg, whereas the reference compound had a slightly higher LD50 value at 2.66 mol/kg. The lowest observed adverse effect level (LOAEL) in rat models was predicted for all the Triphala-derived polyphenols and the reference drug to study the oral chronic toxicity. The LOAEL values for punicalagin, chebulagic acid, and sennoside C were 12.221, 10.72, and 7.099 (log mg/kg_bw/day), respectively (Table 4) (Pires et al., 2015). TJ-M2010-5 exhibited a significantly lower LOAEL of 1.112 compared to the polyphenols, suggesting a higher potential for chronic toxicity. Although these polyphenols are broadly recognized for their anti-inflammatory properties, and numerous *in vitro* and *in vivo* studies have demonstrated their efficacy in inflammatory models, significant limitations still persist. To overcome these challenges, prodrug strategies can be employed to enhance their ADME profiles. Additionally, advanced drug delivery platforms such as nanocarrier-based formulations offer promising approaches to improve the target delivery and bioavailability of the polyphenols, thereby increasing their therapeutic potential (Rautio et al., 2008; Sun et al., 2024).

**TABLE 4:** Drug likeness and pharmacokinetic properties of the top three Triphala-derived polyphenols along with the reference molecule.

Drug likeness and pharmacokinetic properties	Ligands			
	Punicalagin	Chebulagic acid	Sennoside C	TJ-M2010-5
Lipinski’s rule of five	3 Violations	3 Violations	3 Violations	0 Violations
Bioavailability score	0.17	0.11	0.11	0.55
Water solubility (log mol/L)	−2.892	−2.892	−2.892	−3.812
Intestinal absorption (%)	100	93.835	0	91.865
BBB permeability (log BB)^*^	−4.981	−3.889	−2.44	0.287
CYP450 substrate	No	No	No	Yes (CYP2D6 substrate, CYP3A4 substrate)
CYP450 inhibitors	No	No	No	Yes (CYP2D6 inhibitor, CYP3A4 inhibitor)
Total clearance (log mL/min/kg)	−0.246	−2,632	−1.06	0.813
Renal OCT2 substrate	No	No	No	Yes
AMES toxicity	No	No	No	No
Max. Tolerated dose (log mg/kg/day)	0.438	0.438	0.458	0.505
Oral rat acute toxicity (LD50) (mol/kg)	2.482	2.482	2.482	2.66
Oral rat chronic toxicity (LOAEL) (log mg/kg_bw/day)	12.221	10.72	7.099	1.112
Hepatotoxicity	No	No	No	Yes

Note: blood–brain barrier (BBB) permeability is typically expressed as log BB. If the log BB is below −1, the drug is considered to have poor BBB permeability and is unlikely to cross into the central nervous system (CNS), suggesting low therapeutic effects or adverse toxicities in the CNS. To overcome the poor BBB permeability of polyphenols, they can be encapsulated with nano-carrier-based drug delivery systems.

## Discussion

The first line of defense in the human body is provided by the innate immune response, which is a nonspecific protective mechanism that involves numerous signaling pathways. Among these, NF-κB is one of the most important pathways, which involves the adaptor protein MyD88. MyD88 mediates the downstream signaling of the NF-κB pathway, leading to the subsequent release of pro-inflammatory cytokines. MyD88 comprises three domains, namely, the death domain (DD) at the N-terminal, the Toll/interleukin-1 receptor (TIR) domain at the C-terminal, and an intermediary domain (INT) (Ohnishi et al., 2009; Saikh, 2021). The TIR domain (155–296 aa) facilitates MyD88 to interact with the TIR domain of Toll-like receptors (TLRs) or interleukin-1 receptors (IL-1Rs) to form higher-order complexes by recruiting more TIR domains of MyD88 to the vicinity. DD (54–109 aa) activates interleukin-1R (IL-1R)-associated kinase (IRAK) 1–4, subsequently undergoing various phosphorylation procedures and releasing the transcription factor NF-κB. The INT (110–155 aa) domain forms a link between the TIR domain and DD. Studies show that INT is essential for IRAK phosphorylation, and its absence is associated with the inability of MyD88 in downstream signaling (Clabbers et al., 2021). Since the TIR domain of MyD88 plays an important role in the triggered immune response, many synthetic drugs have been used for targeting this domain, which were found to have harmful side effects. Polyphenols are naturally occurring phytochemicals in all plant-based foods, known for their anti-inflammatory properties and toxin-free nature. A search for a toxin-free Ayurvedic drug with significant anti-inflammatory properties has led to the drug named Triphala, which is made from three fruits. These fruits are rich in polyphenols that can work synergistically to provide health benefits, including anti-inflammatory properties. Hence, the 33 major polyphenols were selected, and their crystal structures were optimized with the MMFF94 force field for docking studies with the TIR domain of MyD88. Our docking studies revealed that the polyphenols had good binding affinity toward the TIR domain of the adaptor protein, which was expressed in binding energy. In particular, punicalagin, chebulagic acid, and sennoside C demonstrated the highest binding affinities for the target protein, with more negative scores. In molecular docking, high negative scores indicate efficient binding; however, the interaction, stability, and conformational changes of these complexes were further confirmed by studying their non-covalent bonds and by subjecting them to an MD simulation for 200 ns (Ghosh et al., 2021; Rudrapal et al., 2022). MD simulation is a computational approach that aids in the real-time simulation of the movements of atoms, providing insights into the kinetic and thermodynamic characteristics of biomolecules (Gelpi et al., 2015; Hollingsworth and Dror, 2018). Key integrity parameters, such as RMSD, RMSF, SASA, Rg, and H-bonds, were evaluated for the stability and dynamicity of atoms present in protein–ligand complexes. Notably, in our study, the punicalagin and chebulagic acid complexes exhibited a stable graph of RMSD, SASA, and Rg, while the protein–TJ-M2010-5 and protein–sennoside C complexes demonstrated more fluctuations in the RMSD, SASA, and Rg, suggesting structural instability compared to other complexes and the target protein. In general, the RMSD is a key metric that helps in assessing the stability of molecular structure, and a higher RMSD value indicates less stability. SASA measures the extent of protein exposure to the solvent. A high SASA value indicates that a large portion of the protein is exposed to the solvent, suggesting that the ligands are not properly bound to the protein (Arooj et al., 2020; Ghosh et al., 2021; Rudrapal et al., 2022). Rg provides insights into the compactness and rigidness of atoms in biomolecules. Higher Rg values indicate the increased flexibility of a protein with weaker interactions between the protein and ligand. RMSF provides insights into the atomic-level modifications in the protein chain, which aids in studying the flexibility and stability of residues present in the protein. RMSF has a direct influence on the affinity and specificity of ligand binding. A protein–ligand complex with lower RMSF denotes the strong interaction between them (Arooj et al., 2020; Ghosh et al., 2021; Rudrapal et al., 2022). Our study showed that the RMSF of the reference drug complex had higher fluctuations than the polyphenol complexes. Overall, our MD trajectories demonstrated that the binding of polyphenols increased the stability of the protein structure more than the reference drug. Notably, the strength of ligand binding was governed by the non-covalent hydrogen bonds. Initially, the proteins may display numerous intramolecular H-bonds in their naïve state to stabilize their secondary and tertiary structures. Upon binding of ligand molecules, some of the intramolecular bonds are degraded and replaced by intermolecular H-bonds (Arooj et al., 2020; Ghosh et al., 2021; Rudrapal et al., 2022). Intermolecular H-bonds were quantified after docking and MD simulation. Notably, the H-bond in the punicalagin complex remained the same after docking and MD simulation, whereas the chebulagic acid complex showed increased H-bonds after MD simulation. Additional H-bond formation during MD simulation suggests that the protein–ligand system has explored multiple conformations under dynamic conditions.

The binding free energy was calculated with MM-PBSA, which provided insights into the energy contributed to the binding of the target protein and ligands (Rifai et al., 2019; Murugesan et al., 2021). In general, the higher the binding free energy, the lower the binding affinity. Our findings revealed that the reference drug has a higher binding free energy than the tested polyphenols. Meanwhile, the chebulagic acid and punicalagin complexes displayed very low binding free energies in MM-PBSA, which correlates with the other MD trajectories. Overall, MD simulation data corroborated the findings of molecular docking, suggesting that the binding of punicalagin and chebulagic acid has enhanced the stability and integrity of the target protein, with significant stabilization observed after 145–150 ns, as evidenced by a peak reduction in RMSD, Rg, and SASA values. In addition, evaluation of the drug-likeness and pharmacokinetic properties was essential for knowing the druggability of a therapeutic compound to avoid drug rejection during the clinical trials (Aljarba et al., 2022). Here, the selected polyphenols exhibited limitations in ADME properties, particularly in absorption and metabolism. Unlike the reference compound, the polyphenols have not exhibited hepatotoxicity or mutagenicity. Incorporation of sustained drug delivery systems can improve the pharmacokinetic performance of these polyphenols. However, direct experimental evidence of these polyphenols interacting with the TIR domain of the MyD88 adaptor protein was not conclusively demonstrated. Studies on *in vitro* and *in vivo* inflammatory models concluded that using Triphala extracts can suppress the inflammatory biomarkers, including TNF-α, IL-1β, and IL-6, suggesting the indirect modulation of MyD88-mediated NF-κB expression (Kalaiselvan and Rasool, 2016). Further *in vitro* studies are needed to elucidate the precise mechanism of polyphenol interactions with MyD88. Overall, our findings provide strong evidence for supporting the hypothesis that Triphala-derived polyphenols modulate the triggered immune response by interacting with the TIR domain of MyD88, thereby inhibiting homodimerization, which may further suppress the downstream immune signaling pathways. This result highlights the therapeutic potential of Triphala-derived polyphenols in the management of inflammatory diseases.

## Conclusion

In conclusion, this study employed a computational approach to provide insights into the molecular mechanisms underlying the anti-inflammatory potential of Triphala-derived polyphenols. The findings highlighted the non-toxic nature of polyphenols and their favorable interaction profiles with the TIR domain of the MyD88 adaptor protein. Compounds such as chebulagic acid and punicalagin have shown the maximum interaction with the target protein, which could act as promising scaffolds for drug design. However, to comprehensively evaluate the proposed hypothesis, further *in vitro* investigations using cell lines such as RAW 264.7 or THP-1 are required to validate the mechanism of NF-κB inhibition and cytokine reduction. Additionally, *in vivo* inflammatory models could provide mechanistic insights into the pharmacodynamics of these compounds, thereby supporting the formulation of potential therapeutic agents to combat chronic inflammatory conditions.

## Data Availability

The original contributions presented in the study are included in the article/Supplementary Material; further inquiries can be directed to the corresponding author.

## References

[B1] Abraham M. J. Murtola T. Schulz R. Páll S. Smith J. C. Hess B. (2015). GROMACS: high performance molecular simulations through multi-level parallelism from laptops to supercomputers. SoftwareX 1–2, 19–25. 10.1016/j.softx.2015.06.001

[B2] Aliye M. Dekebo A. Tesso H. Abdo T. Eswaramoorthy R. Melaku Y. (2021). Molecular docking analysis and evaluation of the antibacterial and antioxidant activities of the constituents of Ocimum cufodontii. Sci. Rep. 11, 10101. 10.1038/s41598-021-89557-x 33980935 PMC8115310

[B3] Aljarba N. H. Hasnain M. S. Bin-Meferij M. M. Alkahtani S. (2022). An in-silico investigation of potential natural polyphenols for the targeting of COVID main protease inhibitor. J. King Saud Univ. - Sci. 34, 102214. 10.1016/j.jksus.2022.102214 35811756 PMC9250415

[B4] Arooj M. Shehadi I. Nassab C. N. Mohamed A. A. (2020). Physicochemical stability study of protein–benzoic acid complexes using molecular dynamics simulations. Amino Acids 52, 1353–1362. 10.1007/s00726-020-02897-2 33006112

[B5] Arpornchayanon W. Subhawa S. Jaijoy K. Lertprasertsuk N. Soonthornchareonnon N. Sireeratawong S. (2022). Safety of the oral triphala recipe from acute and chronic toxicity tests in sprague-dawley rats. Toxics 10, 514. 10.3390/toxics10090514 36136479 PMC9503284

[B6] Clabbers M. T. B. Holmes S. Muusse T. W. Vajjhala P. R. Thygesen S. J. Malde A. K. (2021). MyD88 TIR domain higher-order assembly interactions revealed by microcrystal electron diffraction and serial femtosecond crystallography. Nat. Commun. 12, 2578. 10.1038/s41467-021-22590-6 33972532 PMC8110528

[B7] Daina A. Michielin O. Zoete V. (2017). SwissADME: a free web tool to evaluate pharmacokinetics, drug-likeness and medicinal chemistry friendliness of small molecules. Sci. Rep. 7, 42717. 10.1038/srep42717 28256516 PMC5335600

[B8] Di Padova F. Quesniaux V. F. J. Ryffel B. (2018). MyD88 as a therapeutic target for inflammatory lung diseases. Expert Opin. Ther. Targets 22, 401–408. 10.1080/14728222.2018.1464139 29658361

[B9] Eberhardt J. Santos-Martins D. Tillack A. F. Forli S. (2021). AutoDock Vina 1.2.0: new docking methods, expanded force field, and Python bindings. J. Chem. Inf. Model. 61, 3891–3898. 10.1021/acs.jcim.1c00203 34278794 PMC10683950

[B10] Gelpi J. Hospital A. Goñi R. Orozco M. (2015). Molecular dynamics simulations: advances and applications. AABC 37, 37. 10.2147/AABC.S70333 PMC4655909 26604800

[B11] Ghosh R. Chakraborty A. Biswas A. Chowdhuri S. (2021). Evaluation of green tea polyphenols as novel corona virus (SARS CoV-2) main protease (Mpro) inhibitors – an *in silico* docking and molecular dynamics simulation study. J. Biomol. Struct. Dyn. 39, 4362–4374. 10.1080/07391102.2020.1779818 32568613 PMC7332865

[B12] Hollingsworth S. A. Dror R. O. (2018). Molecular dynamics simulation for all. Neuron 99, 1129–1143. 10.1016/j.neuron.2018.08.011 30236283 PMC6209097

[B13] Hou T. Wang J. Li Y. Wang W. (2011). Assessing the performance of the MM/PBSA and MM/GBSA methods. 1. The accuracy of binding free energy calculations based on molecular dynamics simulations. J. Chem. Inf. Model. 51, 69–82. 10.1021/ci100275a 21117705 PMC3029230

[B14] Hu S. Li S. Xu Y. Huang X. Mai Z. Chen Y. (2024). The antitumor effects of herbal medicine Triphala on oral cancer by inactivating PI3K/Akt signaling pathway: based on the network pharmacology, molecular docking, *in vitro* and *in vivo* experimental validation. Phytomedicine 128, 155488. 10.1016/j.phymed.2024.155488 38493718

[B15] Kalaiselvan S. Rasool M. K. (2016). Triphala herbal extract suppresses inflammatory responses in LPS-stimulated RAW 264.7 macrophages and adjuvant-induced arthritic rats via inhibition of NF-κB pathway. J. Immunotoxicol. 13, 509–525. 10.3109/1547691X.2015.1136010 27438966

[B16] Kumari R. Kumar R. Open Source Drug Discovery Consortium Lynn A. (2014). g_mmpbsa —a GROMACS tool for high-throughput MM-PBSA calculations. J. Chem. Inf. Model. 54, 1951–1962. 10.1021/ci500020m 24850022

[B17] Laskowski R. A. Swindells M. B. (2011). LigPlot+: multiple ligand–protein interaction diagrams for drug discovery. J. Chem. Inf. Model. 51, 2778–2786. 10.1021/ci200227u 21919503

[B18] Lipinski C. A. (2004). Lead- and drug-like compounds: the rule-of-five revolution. Drug Discov. Today Technol. 1, 337–341. 10.1016/j.ddtec.2004.11.007 24981612

[B19] Loiarro M. Capolunghi F. Fantò N. Gallo G. Campo S. Arseni B. (2007). Pivotal Advance: inhibition of MyD88 dimerization and recruitment of IRAK1 and IRAK4 by a novel peptidomimetic compound. J. Leukoc. Biol. 82, 801–810. 10.1189/jlb.1206746 17548806

[B20] Loiarro M. Sette C. Gallo G. Ciacci A. Fantò N. Mastroianni D. (2005). Peptide-mediated interference of TIR domain dimerization in MyD88 inhibits interleukin-1-dependent activation of NF-κB. J. Biol. Chem. 280, 15809–15814. 10.1074/jbc.C400613200 15755740

[B21] Martin Y. C. (2005). A bioavailability score. J. Med. Chem. 48, 3164–3170. 10.1021/jm0492002 15857122

[B22] Murugesan S. Kottekad S. Crasta I. Sreevathsan S. Usharani D. Perumal M. K. (2021). Targeting COVID-19 (SARS-CoV-2) main protease through active phytocompounds of ayurvedic medicinal plants – emblica officinalis (Amla), Phyllanthus niruri Linn. (Bhumi Amla) and Tinospora cordifolia (Giloy) – a molecular docking and simulation study. Comput. Biol. Med. 136, 104683. 10.1016/j.compbiomed.2021.104683 34329860 PMC8302490

[B23] Ohnishi H. Tochio H. Kato Z. Orii K. E. Li A. Kimura T. (2009). Structural basis for the multiple interactions of the MyD88 TIR domain in TLR4 signaling. Proc. Natl. Acad. Sci. U.S.A. 106, 10260–10265. 10.1073/pnas.0812956106 19506249 PMC2693180

[B24] Olaokun O. O. Manonga S. A. Zubair M. S. Maulana S. Mkolo N. M. (2022). Molecular docking and molecular dynamics studies of antidiabetic phenolic compound isolated from leaf extract of englerophytum magalismontanum (sond.) T.D.Penn. Molecules 27, 3175. 10.3390/molecules27103175 35630652 PMC9145638

[B25] Olson M. A. Lee M. S. Kissner T. L. Alam S. Waugh D. S. Saikh K. U. (2015). Discovery of small molecule inhibitors of MyD88-dependent signaling pathways using a computational screen. Sci. Rep. 5, 14246. 10.1038/srep14246 26381092 PMC4585646

[B26] Pires D. E. V. Blundell T. L. Ascher D. B. (2015). pkCSM: predicting small-molecule pharmacokinetic and toxicity properties using graph-based signatures. J. Med. Chem. 58, 4066–4072. 10.1021/acs.jmedchem.5b00104 25860834 PMC4434528

[B27] Prasad S. Srivastava S. K. (2020). Oxidative stress and cancer: chemopreventive and therapeutic role of triphala. Antioxidants 9, 72. 10.3390/antiox9010072 31941067 PMC7022920

[B28] Rautio J. Kumpulainen H. Heimbach T. Oliyai R. Oh D. Järvinen T. (2008). Prodrugs: design and clinical applications. Nat. Rev. Drug Discov. 7, 255–270. 10.1038/nrd2468 18219308

[B29] Rifai E. A. Van Dijk M. Vermeulen N. P. E. Yanuar A. Geerke D. P. (2019). A comparative linear interaction energy and MM/PBSA study on SIRT1–ligand binding free energy calculation. J. Chem. Inf. Model. 59, 4018–4033. 10.1021/acs.jcim.9b00609 31461271 PMC6759767

[B30] Rudrapal M. Celik I. Khan J. Ansari M. A. Alomary M. N. Alatawi F. A. (2022). Identification of bioactive molecules from Triphala (Ayurvedic herbal formulation) as potential inhibitors of SARS-CoV-2 main protease (Mpro) through computational investigations. J. King Saud Univ. - Sci. 34, 101826. 10.1016/j.jksus.2022.101826 35035181 PMC8744360

[B31] Saikh K. U. (2021). MyD88 and beyond: a perspective on MyD88-targeted therapeutic approach for modulation of host immunity. Immunol. Res. 69, 117–128. 10.1007/s12026-021-09188-2 33834387 PMC8031343

[B32] Shanmuganathan S. Angayarkanni N. (2018). Chebulagic acid Chebulinic acid and Gallic acid, the active principles of Triphala, inhibit TNFα induced pro-angiogenic and pro-inflammatory activities in retinal capillary endothelial cells by inhibiting p38, ERK and NFkB phosphorylation. Vasc. Pharmacol. 108, 23–35. 10.1016/j.vph.2018.04.005 29678603

[B33] Song J. Chen D. Pan Y. Shi X. Liu Q. Lu X. (2021). Discovery of a novel MyD88 inhibitor M20 and its protection against sepsis-mediated acute lung injury. Front. Pharmacol. 12, 775117. 10.3389/fphar.2021.775117 34912226 PMC8666603

[B34] Sun Q. Lv M. Li Y. (2024). Nanotechnology-based drug delivery systems for curcumin and its derivatives in the treatment of cardiovascular diseases. J. Funct. Foods 122, 106476. 10.1016/j.jff.2024.106476

[B35] Tarasiuk A. Mosińska P. Fichna J. (2018). Triphala: current applications and new perspectives on the treatment of functional gastrointestinal disorders. Chin. Med. 13, 39. 10.1186/s13020-018-0197-6 30034512 PMC6052535

[B36] Vanommeslaeghe K. Hatcher E. Acharya C. Kundu S. Zhong S. Shim J. (2010). CHARMM general force field: a force field for drug‐like molecules compatible with the CHARMM all‐atom additive biological force fields. J. Comput. Chem. 31, 671–690. 10.1002/jcc.21367 19575467 PMC2888302

[B37] Vieira I. H. P. Botelho E. B. De Souza Gomes T. J. Kist R. Caceres R. A. Zanchi F. B. (2023). Visual dynamics: a WEB application for molecular dynamics simulation using GROMACS. BMC Bioinforma. 24, 107. 10.1186/s12859-023-05234-y PMC10031864 36949402

[B38] Wang J. Q. Jeelall Y. S. Ferguson L. L. Horikawa K. (2014). Toll-like receptors and cancer: MYD88 mutation and inflammation. Front. Immunol. 5, 367. 10.3389/fimmu.2014.00367 25132836 PMC4116802

[B39] Wang W. Ige O. O. Ding Y. He M. Long P. Wang S. (2023). Insights into the potential benefits of triphala polyphenols toward the promotion of resilience against stress-induced depression and cognitive impairment. Curr. Res. Food Sci. 6, 100527. 10.1016/j.crfs.2023.100527 37377497 PMC10291000

[B40] Xie L. Jiang F.-C. Zhang L.-M. He W.-T. Liu J.-H. Li M.-Q. (2016). Targeting of MyD88 homodimerization by novel synthetic inhibitor TJ-m2010-5 in preventing colitis-associated colorectal cancer. JNCI.J. 108, djv364. 10.1093/jnci/djv364 26712311

[B41] Zhang S.-S. Liu M. Liu D.-N. Shang Y.-F. Wang Y.-H. Du G.-H. (2022). ST2825, a small molecule inhibitor of MyD88, suppresses NF-κB activation and the ROS/NLRP3/Cleaved caspase-1 signaling pathway to attenuate lipopolysaccharide-stimulated neuroinflammation. Molecules 27, 2990. 10.3390/molecules27092990 35566338 PMC9106063

[B42] Zhao Y. H. Abraham M. H. Le J. Hersey A. Luscombe C. N. Beck G. (2002). Rate-limited steps of human oral absorption and QSAR studies. Pharm. Res. 19, 1446–1457. 10.1023/A:1020444330011 12425461

[B43] Zhivkova Z. Doytchinova I. (2013). Quantitative structure – clearance relationships of acidic drugs. Mol. Pharm. 10, 3758–3768. 10.1021/mp400251k 23898951

[B44] Zou Z. Shang R. Zhou L. Du D. Yang Y. Xie Y. (2023). The novel MyD88 inhibitor TJ-m2010-5 protects against hepatic ischemia-reperfusion injury by suppressing pyroptosis in mice. Transplantation 107, 392–404. 10.1097/TP.0000000000004317 36226835 PMC9875839

