# Supplementary material for: In silico analysis of Triphala-derived polyphenols as inhibitors of TIR–TIR homodimerization in the inflammatory pathway
Source: Front Bioinform. 2025 May 29;5:1565700. doi: 10.3389/fbinf.2025.1565700 (PMC12158960; doi:10.3389/fbinf.2025.1565700)
Supplement: Supplementary file 1 [file Table1.docx]

**Supplementary data**

**Table 1.** Binding affinity or docking scores of different Triphala polyphenenols and the reference compound with TIR-domain of MyD88 protein.

**Note:** More negative the docking score, higher the binding affinity for the ligand with the target protein.

| **S.No** | **Ligands** | **Docking score/Binding affinity(kcal/mol)** |
| --- | --- | --- |
| 1 | Arjunolic acid | -7.1 |
| 2 | Ascorbic acid | -4.9 |
| 3 | Betasitosterol | -6.5 |
| 4 | Brevifolin | -5.1 |
| 5 | **Chebulagic acid** | **-8.5** |
| 6 | Chebulic acid | -6.8 |
| 7 | Chebulinic acid | -7.8 |
| 8 | Corilagin | -7.2 |
| 9 | Ellagic acid | -7.1 |
| 10 | Emblicanin A | -7.8 |
| 11 | Emblicanin B | -8 |
| 12 | Epigallocatechin | -6.7 |
| 13 | Gallocatechin | -6.8 |
| 14 | Kaempferol | -7.3 |
| 15 | Luteolin | -7.9 |
| 16 | Maslinic acid | -7.4 |
| 17 | Melissic acid | -4.4 |
| 18 | Myristic acid | -4.5 |
| 19 | Palmitic acid | -3.9 |
| 20 | Pentagalloylglucose | -8.1 |
| 21 | Phyllaemblicin A | -7.6 |
| 22 | Phyllaemblicin B | -7.2 |
| 23 | Phyllaemblicin C | -7.3 |
| 24 | Phyllanthin | -5.1 |
| 25 | Progallin A | -6 |
| 26 | Punicafolin | -7.6 |
| 27 | **Punicalagin** | **-9.1** |
| 28 | Quercetin | -7.6 |
| 29 | Quinic acid | -5.1 |
| 30 | **Sennoside C** | **-8.4** |
| 31 | Sennoside E | -8.2 |
| 32 | Syringic acid | -5.1 |
| 33 | Tannic acid | -6.3 |
| 34 | **TJ-M2010-5** | **-7.4** |
